# Evaluation of Baseline Investigations for First-Contact Patients Receiving Psychotropic Treatment at a Tertiary Facility in South-West Nigeria: A Two-Year Clinical Audit

**DOI:** 10.7759/cureus.111581

**Published:** 2026-06-26

**Authors:** Ayodeji S Erubu, Miracle M Oluwasanmi, Michael N Nguh, Oluwabunmi N Buhari, Precious Olarotimi, Olaniyi A Olatunde, Anas I Yakubu, Ayobola O Adebowale, Blessing T Aguda

**Affiliations:** 1 Psychiatry, Afe Babalola University, Ado-Ekiti, NGA; 2 Behavioral Sciences, University of Ilorin, Ilorin, NGA; 3 Anatomic Pathology and Forensic Medicine, Afe Babalola University, Ado-Ekiti, NGA; 4 Psychiatry and Behavioral Sciences, Federal Teaching Hospital, Birnin Kebbi, NGA; 5 Pediatrics and Child Health, Lesha Hospital, Abuja, NGA; 6 Family Medicine, Afe Babalola University, Ado-Ekiti, NGA

**Keywords:** baseline investigations, clinical audit, psychiatric services, psychotropic medications, quality improvement

## Abstract

Introduction

Baseline investigations are recommended before initiating psychotropic medications to identify pre-existing medical conditions and reduce treatment-related adverse effects. This audit assessed compliance with recommended baseline investigations among first-contact patients receiving psychotropic treatment at a tertiary hospital in South-West Nigeria.

Materials and methods

A retrospective clinical audit was conducted using electronic medical records (EMRs) of first-contact patients managed between January 2024 and December 2025. Audit standards were derived from the National Institute for Health and Care Excellence (NICE) and the Royal College of Psychiatrists (RCPsych) recommendations. Compliance was categorized as high (≥75%), moderate (50%-74%), or low (<50%). Data were analyzed using descriptive statistics, chi-square tests, independent-samples t-tests, and one-way analysis of variance (ANOVA).

Results

Of 345 first-contact patients reviewed, 262 (75.9%) received at least one psychotropic medication and were included in the audit. Substance use disorders (61, 23.28%) and depressive episodes (60, 22.90%) were the most common diagnoses. Compliance with weight/body mass index (BMI) assessment, vital signs, and movement disorder screening was 100%. Compliance with laboratory and cardiac investigations was substantially lower: electrolytes/urea/creatinine (E/U/Cr) (25.2%), full blood count (FBC) (22.5%), fasting glucose/glycated hemoglobin (HbA1c) (19.6%), electrocardiography (11.4%), liver function tests (LFTs) (8.8%), and fasting lipid profile (FLP) (7.1%). No patient completed baseline prolactin testing. Overall, 180 patients (68.7%) had low compliance. Inpatients demonstrated significantly higher compliance than outpatients (55.5% versus 34.8%; p < 0.001).

Conclusions

Compliance with recommended baseline investigations was suboptimal, particularly for laboratory and electrocardiogram (ECG) monitoring. Local monitoring protocols, improved access to investigations, and regular re-audit may improve adherence to recommended standards.

## Introduction

Psychotropic treatment refers to the use of medications that act on the central nervous system to treat mental and behavioral disorders by influencing neurotransmitter systems involved in mood, cognition, perception, and behavior. Unlike most medications that primarily target peripheral organ systems, psychotropic medications exert their therapeutic effects mainly through actions on brain function and neural signalling pathways [1,2]. Major classes of psychotropic medications include antipsychotics, antidepressants, mood stabilizers, anxiolytics, and psychostimulants [2].

Psychotropic treatments remain the mainstay for managing a wide range of psychiatric disorders, including schizophrenia, bipolar disorder, depression, anxiety disorders, and substance use disorders. While these treatments are highly effective in symptom control and relapse prevention, many are associated with significant metabolic (weight gain, diabetes, and dyslipidemia), cardiovascular (QT prolongation and arrhythmias), neurological (extrapyramidal symptoms), endocrine (hyperprolactinemia), hepatic (drug-induced liver injury), and hematological (agranulocytosis and leukopenia) adverse effects, necessitating comprehensive baseline physical health assessment and ongoing monitoring to maintain quality and safe psychiatric practice [1-4].

Individuals living with mental illness have also been reported to have increased risk of comorbidities, both physical and secondary psychiatric comorbidities, when compared to the general population [5]. The most frequently reported comorbidities include hypertension, diabetes mellitus, dyslipidemia, cardiac arrhythmias, thyroid dysfunction, and renal impairment [5]. Adverse effects of psychotropic prescriptions often contribute to these comorbidities and consequently contribute to premature mortality and increased morbidity observed in patients receiving psychiatric care [6].

To mitigate these risks, several international bodies, including the National Institute for Health and Care Excellence (NICE) and the Royal College of Psychiatrists (RCPsych), have developed evidence-based recommendations for baseline and periodic physical and laboratory investigations before and during psychotropic treatment [7,8]. These recommendations typically include the assessment of body mass index (BMI), blood pressure, fasting glucose or glycated hemoglobin (HbA1c), lipid profile, liver function test (LFT) and renal function test, full blood count (FBC), electrocardiography, prolactin levels, and pregnancy testing where indicated [7,8]. Appropriate baseline investigations facilitate the early identification of pre-existing medical conditions, guide medication selection, and reduce treatment-related morbidity and mortality [9].

Despite the existence of these guidelines, studies across different healthcare settings consistently demonstrate suboptimal compliance with recommended monitoring practices [10,11]. Although much of the published evidence originates from high-income countries such as the United Kingdom, the barriers to compliance may be more pronounced in low- and middle-income countries (LMICs) due to limited resources, poor health financing, inadequate laboratory infrastructure, and the poor integration of physical and mental healthcare services [11]. In Nigeria and many other sub-Saharan African countries, available evidence suggests that physical health assessment among psychiatric patients is often inconsistent and may receive less attention compared with the management of primary psychiatric symptoms. As a result, psychotropic treatment-related adverse effects and comorbid medical conditions may remain undetected, increasing the risk of preventable morbidity [5,12].

Nonetheless, there remains limited evidence from routine clinical practice in LMICs, including Nigeria, assessing adherence to guideline-recommended baseline investigations among patients receiving psychotropic medications [10-12]. This clinical audit therefore aimed to evaluate compliance with established guideline recommendations for baseline investigations among patients receiving psychotropic treatment at Afe Babalola University Multi-System Hospital (AMSH) over a two-year period.

## Materials and methods

Study design and setting

This study is a retrospective clinical audit conducted at the Mental Health Department of Afe Babalola University Multi-System Hospital (AMSH). AMSH is a tertiary teaching hospital providing psychiatric and multidisciplinary healthcare services in South-West Nigeria [13]. The audit evaluates compliance with recommended baseline physical and laboratory investigations among patients receiving psychotropic treatment.

Study population

The study population comprised all eligible patients who received psychiatric care at the Mental Health Department of the facility between January 1, 2024, and December 31, 2025, using a total population sampling technique. Patients were eligible for inclusion if they were managed for the first time in either the psychiatric outpatient or inpatient units, received pharmacological treatment for a psychiatric condition, and had accessible electronic medical records (EMRs) available for review. While patients with incomplete clinical records were generally excluded, the following categories were additionally excluded because they did not represent first-contact cases: outpatients admitted following relapse, readmission cases, and individuals who received psychiatric care while admitted to other units of the hospital for primarily nonpsychiatric conditions.

Data collection

Data were manually extracted from the hospital’s electronic medical record (EMR) system between March and April 2026 using a structured data collection form and subsequently entered into a Microsoft Excel (Microsoft Corp., Redmond, WA) database for analysis. Information collected included socio-demographic characteristics, psychiatric diagnoses (diagnoses were established using the International Classification of Diseases, Tenth Revision {ICD-10} or the Diagnostic and Statistical Manual of Mental Disorders, Fifth Edition {DSM-5}, where applicable), medical comorbidities, prescribed psychotropic medications, and the documentation of baseline and follow-up physical and laboratory investigations [14,15].

Audit standards

The audit standards were developed based on clinical practice guidelines issued by the National Institute for Health and Care Excellence (NICE) and the Royal College of Psychiatrists (RCPsych) [7,8]. These guidelines were used to develop the list of recommended baseline physical and laboratory investigations for each psychotropic medication and the clinical audit scoring system. Where recommendations were conditional, medication-specific, or not explicitly stated, the study team adapted the audit criteria by consensus to reflect routine psychiatric practice within the local setting.

Outcome measures

The primary outcome of this study was compliance with recommended baseline physical and laboratory investigations among patients receiving psychotropic medications.

Compliance scoring system

Compliance with each recommended investigation was assessed using a binary scoring system. A score of 1 was assigned when an investigation had been completed or when a clinically appropriate reason for its omission was documented in the patient’s medical record. A score of 0 was assigned when the investigation had not been completed and no justification for omission was documented.

For each patient, an overall compliance percentage was calculated using the following formula: compliance percentage = (total score obtained/maximum obtainable score) × 100.

Compliance scores were further categorized into three levels: high compliance (≥75%), moderate compliance (50%-74%), and low compliance (<50%). These categories were used to interpret adherence to recommended baseline investigation standards for all patients under study, as previously described in similar studies [9,16].

Data analysis

Data were analyzed using the Statistical Package for Social Sciences (SPSS) version 29.0 (IBM Corp., Armonk, NY). Descriptive statistics were used to summarize patient characteristics and compliance outcomes. Categorical variables were presented as frequencies and percentages, while continuous variables were summarized using means and standard deviations (SD). Assumptions of normality and homogeneity of variances were assessed prior to the use of parametric tests. Comparisons of mean compliance scores between groups were performed using independent-samples t-tests or one-way analysis of variance (ANOVA). Differences in compliance categories (high, moderate, and low) between groups were assessed using the chi-square test. Statistical significance was set at p < 0.05. Findings were presented using appropriate tables and a bar chart.

Audit benchmark

The audit benchmark was defined as 100% compliance with recommended baseline investigations. Accordingly, all patients were expected to have completed the full set of recommended baseline investigations (based on psychotropic medications they were placed on) or to have a documented clinical justification for any omitted investigation.

Ethical considerations

Approval for this study was obtained from the Research and Ethics Committee of Afe Babalola University Multi-System Hospital, with approval number: AMSH/REC/26/62/002. Patient confidentiality was maintained by anonymizing all extracted data.

## Results

A total of 345 patients received psychiatric (pharmacological and non-pharmacological) care for the first time between January 2024 and December 2025. Of these, 262 (75.94%) met the inclusion criteria and were evaluated in this study.

Socio-demographic and clinical characteristics of first-contact patients receiving psychotropic medications

The mean age of participants reviewed for this audit was 34.35 ± 17.15 years, with the majority aged 18-44 years (192, 73.28%). Women constituted 135 (51.53%) of the sample, while men accounted for 127 (48.47%) (Table 1). Sixty-one (23.28%) had a diagnosis of substance use disorders, 60 (22.90%) were diagnosed with depressive episode, 36 (13.74%) were managed for anxiety disorders, and 37 (14.12%) had a psychotic disorder or schizophrenia. A smaller proportion had sleep disorder (primary insomnia) (18, 6.87%), somatoform disorder (17, 6.49%), bipolar affective disorder (four, 1.53%), and other less common diagnoses. Psychiatric or medical comorbidities were documented in 59 (22.52%) of all participants (Table 2).

**Table 1:** Socio-demographic Characteristics (N = 262) Mean (SD) age: 34.35 (17.15) years SD, standard deviation; N/A, not available

Variable	Frequency (n)	Percentage (%)
Age group		
<18 years	7	2.67%
18-44 years	192	73.28%
45-64 years	44	16.79%
>65 years	19	7.25%
Gender		
Male	127	48.47%
Female	135	51.53%
Religion		
Christianity	233	88.93%
Islam	25	9.54%
N/A/undisclosed	4	1.53%
Occupation		
Employed	97	37.02%
Unemployed	38	14.50%
Retired	10	3.82%
Students	117	44.66%

**Table 2:** Clinical Variables (N = 262)

Primary Diagnosis	Frequency (n)	Percentage (%)
Substance use disorders	61	23.28%
Depressive episode	60	22.90%
Psychotic disorder	37	14.12%
Anxiety disorders	36	13.74%
Primary insomnia	18	6.87%
Somatoform disorder	17	6.49%
Organic mental disorder	5	1.91%
Bipolar affective disorder (BAD)	4	1.53%
Conversion disorders	4	1.53%
Acute stress reaction/disorder	4	1.53%
Post-traumatic stress disorder (PTSD)	3	1.15%
Adjustment disorder	3	1.15%
Abnormal grief	2	0.76%
Impulse control disorder	2	0.76%
Seizure disorder	1	0.38%
Obsessive-compulsive disorder	1	0.38%
Sexual dysfunction	1	0.38%
Movement disorder	2	0.76%
Intermittent explosive disorder	1	0.38%
Comorbidity		
No	203	77.48%
Yes	59	22.52%

Compliance with recommended baseline investigations

No documented clinical justifications for the omission of recommended baseline investigations were identified in the reviewed records; therefore, compliance scores reflected completed investigations only. Table 3 shows the varying degree of compliance with recommended baseline investigations observed among first-contact patients receiving psychotropic medications. The assessment of weight/body mass index (BMI), vital signs (blood pressure, pulse, and respiratory rate), and movement disorders revealed 100% compliance with the audit standard. Among laboratory investigations, electrolytes/urea/creatinine (E/U/Cr) testing had the highest compliance rate (66, 25.19%), followed by full blood count (FBC) (59, 22.52%), and fasting glucose/HbA1c (36, 19.57%). All 262 patients receiving psychotropic medication required liver function tests (LFTs) at baseline; of these, only 23 (8.78%) completed this investigation. While seven (7.14%) of 98 requiring fasting lipid profiles (FLP) fully complied with audit standards, there was no documented evidence of serum prolactin being done or evidence of omission in the 95 patients indicated for prolactin testing. However, urine drug testing (UDT) was carried out in 58 (66.7%) of the 87 patients with an indication (Table 3).

**Table 3:** Compliance With Recommended Baseline Investigations Among First-Contact Patients Receiving Psychotropic Treatment (N = 262) Vital signs: blood pressure, pulse, and respiratory rate AMD, assessment of movement disorder; BMI, body mass index; HbA1c, glycated hemoglobin

Investigation	Indicated (n)	Completed (n)	Compliance (%)
Full blood count	262	59	22.52
Electrolytes/urea/creatinine	262	66	25.19
Liver function tests	262	23	8.78
Fasting glucose/HbA1c	184	36	19.57
Fasting lipid profile	98	7	7.14
Prolactin	95	0	0.00
Electrocardiogram (ECG)	237	27	11.39
BMI/weight	262	262	100.00
Vitals signs	262	262	100.00
Brain imaging (MRI/CT)	1	1	100.00
Electroencephalogram (EEG)	1	1	100.00
Urine drug testing (UDT)	87	58	66.67
AMD	262	262	100.00

Patient-level compliance

**Figure 1:**
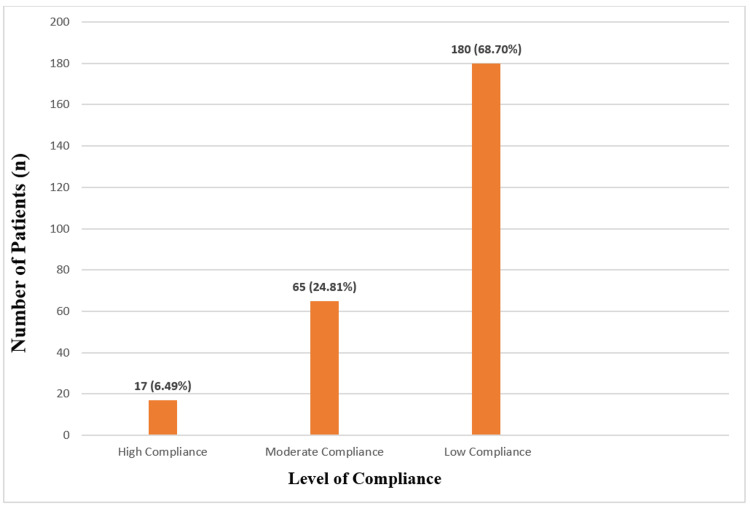
Distribution of Patient-Level Compliance Categories Among Patients Receiving Psychotropic Medications (N = 262) Compliance was classified as high (≥75%), moderate (50%-74%), and low (<50%)

Figure 1 shows the distribution of patient-level compliance categories. While there was predominantly low compliance (180, 68.70%) among first-contact patients receiving psychotropic medications, 65 (24.81%) had moderate compliance, and only a small proportion (17, 6.49%) achieved high compliance with baseline investigations.

Compliance by psychotropic medication class

Patients receiving antipsychotic monotherapy demonstrated the highest mean compliance score (50.36% ± 18.5), whereas those receiving Benzodiazepines as monotherapy had the lowest mean compliance (17.00% ± 24.2). There was a statistically significant difference in the distribution of compliance categories across medication classes (χ² = 13.25; degrees of freedom {df} = 4; p = 0.010), and mean compliance scores also differed significantly between medication classes (F = 10.06; p < 0.001) (Table 4). In addition, Table 5 presents the distribution of specific psychotropic medications prescribed to first-contact patients.

**Table 4:** Compliance by Class of Psychotropic Medications (N = 262) Statistical comparison showed a significant difference in compliance categories across medication classes. For chi-square analysis, compliance was categorized as high/moderate versus low, resulting in a 5 × 2 contingency table (χ² = 13.25; df = 4; p = 0.010). Mean compliance scores also differed significantly between groups (one-way ANOVA: F(4, 257) = 10.06; p < 0.001) df, degrees of freedom; ANOVA, analysis of variance; SD, standard deviation

Medication Class	High, n (%)	Moderate, n (%)	Low, n (%)	Total (n)	Mean compliance (%) ± SD
Antipsychotic monotherapy	3 (7.5%)	18 (45.0%)	19 (47.5%)	40	50.36 ± 18.5
Mood stabilizer monotherapy	2 (20.0%)	0 (0.0%)	8 (80.0%)	10	37.33 ± 29.0
Antidepressant monotherapy	3 (4.4%)	12 (17.6%)	53 (77.9%)	68	39.25 ± 14.1
Benzodiazepine monotherapy	0 (0.0%)	5 (20.0%)	20 (80.0%)	25	17.00 ± 24.2
Combination therapy	9 (7.6%)	30 (25.2%)	80 (67.2%)	119	41.88 ± 19.2

**Table 5:** Distribution of Prescribed Psychotropic Medications Among First-Contact Patients (N = 262) N = total number of first-contact patients who received at least one medication. n = number of first-contact patients prescribed a particular medication, either as monotherapy or in combination with other medications; therefore, patients may be represented in more than one medication category *Medications not classified under the major psychotropic groups but prescribed alongside these agents were categorized as “adjunct” medications

Prescribed Medication	Major Psychotropic Group	Number Treated (n)	Percentage (%) (N = 262)
Amitriptyline	Antidepressant	62	23.66%
Escitalopram	Antidepressant	6	2.29%
Fluoxetine	Antidepressant	36	13.74%
Paroxetine	Antidepressant	2	0.76%
Sertraline	Antidepressant	36	13.74%
Aripiprazole	Antipsychotic	2	0.76%
Chlorpromazine	Antipsychotic	26	9.92%
Clozapine	Antipsychotic	1	0.38%
Flupentixol	Antipsychotic	7	2.67%
Fluphenazine	Antipsychotic	11	4.20%
Haloperidol	Antipsychotic	24	9.16%
Olanzapine	Antipsychotic	17	6.49%
Quetiapine	Antipsychotic	1	0.38%
Risperidone	Antipsychotic	33	12.60%
Trifluoperazine	Antipsychotic	4	1.53%
Carbamazepine	Mood stabilizer	32	12.21%
Sodium valproate	Mood stabilizer	3	1.15%
Bromazepam	Benzodiazepine	1	0.38%
Diazepam	Benzodiazepine	29	11.07%
Nitrazepam	Benzodiazepine	64	24.43%
Benzhexol (trihexyphenidyl)*	Adjunct	13	4.96%
Biopentin (gabapentin)*	Adjunct	1	0.38%
Cognitol (vinpocetine)*	Adjunct	4	1.53%
Dihydrocodeine*	Adjunct	1	0.38%
Donepezil*	Adjunct	1	0.38%
Melatonin*	Adjunct	2	0.76%
Neurovite*	Adjunct	1	0.38%
Vitamin B complex*	Adjunct	7	2.67%
Zopiclone*	Adjunct	3	1.15%

Compliance by treatment setting

Patients admitted at first contact with psychiatric care recorded a higher mean compliance compared to those who were managed as outpatients at first contact (55.50% ± 20.34 versus 34.81% ± 17.85), with a statistically significant difference (t = 7.32; p < 0.001). A significant difference was also observed in the distribution of compliance categories between both groups (χ² = 54.59; df = 2; p < 0.001) (Table 6).

**Table 6:** Compliance Comparison by Treatment Setting (N = 262) There was a significant difference in compliance categories between treatment settings (chi-square test: χ² = 54.59; df = 2; p < 0.001). Mean compliance scores also differed significantly between groups (independent-samples t-test: t = 7.32; p < 0.001) SD: standard deviation

Treatment Setting	High, n (%)	Moderate, n (%)	Low, n (%)	Mean Compliance (%) ± SD
Outpatient (n = 197)	6 (3.05%)	32 (16.24%)	159 (80.71%)	34.81 ± 17.85
Inpatient (n = 65)	11 (16.92%)	33 (50.77%)	21 (32.31%)	55.50 ± 20.34

Comparison of compliance by year

The number of patients recording high compliance increased from three (2.8%) in 2024 to 14 (9.1%) in 2025, while the mean compliance score increased from 37.41% ± 18.93 to 41.72% ± 21.43. However, this improvement was not statistically significant (t = 1.68; p = 0.094). Similarly, the distribution of compliance categories did not differ significantly between the two years (χ² = 4.23; df = 2; p = 0.120). Low compliance remained the predominant category in both years (Table 7).

**Table 7:** Yearly Comparison of Compliance (N = 262) There was no statistically significant difference in compliance categories between 2024 and 2025 (chi-square test: χ² = 4.23; df = 2; p = 0.120). Similarly, there was no significant difference in mean compliance scores between the two years (independent-samples t-test: t = 1.68; p = 0.094) SD: standard deviation

Year	High, n (%)	Moderate, n (%)	Low, n (%)	Mean Compliance (%) ± SD
2024 (n = 108)	3 (2.8%)	27 (25.0%)	78 (72.2%)	37.41 ± 18.93
2025 (n = 154)	14 (9.1%)	38 (24.7%)	102 (66.2%)	41.72 ± 21.43

## Discussion

This clinical audit evaluated compliance with recommended baseline physical and laboratory investigations among patients receiving psychotropic medications who presented for the first time at the Mental Health Department of a tertiary facility in South-West Nigeria.

While basic physical assessments such as weight, vital signs, and movement disorder screening achieved full compliance, laboratory and cardiac investigations were inconsistently performed. The 100% compliance documented for BMI/weight measurement, vital signs, and the assessment of movement disorders suggests that the study facility already runs a system that supports routine physical health assessment as part of psychiatric care. This finding is consistent with the growing recognition that physical examination and clinical assessment should form an integral component of psychiatric practice and are often more readily incorporated into routine care than laboratory-based monitoring [17]. Urine drug testing (UDT) had a compliance rate of 66.7%, the second highest among monitored investigations. Despite being indicated in 87 patients, UDT’s completion rate may reflect its targeted use in those evaluated for substance use disorders.

In sharp contrast, glucose monitoring (fasting glucose/HbA1c) (19.57%), FBC (22.52%), E/U/Cr (25.19%), LFT (8.78%), and FLP (7.14%) were markedly underutilized despite their clear indications and importance in patients initiating psychotropic treatment [1,5]. The particularly low compliance rates for LFT and FLP are concerning, given their critical role in monitoring patients receiving antipsychotics, mood stabilizers, and other psychotropic medications. The liver metabolizes many antipsychotics via the cytochrome P450 (CYP) system and other hepatic pathways, making it vulnerable to drug-induced injury; inadequate monitoring may therefore delay the detection of hepatotoxicity [2]. Similarly, fasting lipid profile (FLP) monitoring is essential, as several second-generation antipsychotics are associated with dyslipidemia, including elevated total cholesterol and other metabolic disturbances [6]. Furthermore, electrocardiography, indicated in 237 patients, was completed in only 11.39%, despite reports linking QT interval prolongation to several antipsychotics and tricyclic antidepressants [18].

In keeping with our findings, a clinical audit conducted among 35 patients on antipsychotics within a medium-secure forensic inpatient unit reported that the monitoring of physical health was sub-par, with the majority of patients not up to date with blood tests or ECGs as per NICE guidelines [11]. Additionally, a study from other low- and middle-income settings reported that structural and financial constraints frequently limit adherence to required investigations [19]. In the present study, most patients paid out of pocket for investigations, which likely influenced both doctors’ patterns of requesting investigations and patient uptake. Financial constraints may therefore have contributed to selective baseline screening [10,19].

Conversely, in settings with extensive health insurance coverage, financial barriers may have a less pronounced effect on compliance with recommended monitoring. For example, a UK study assessing adherence to antipsychotic prescribing and monitoring guidelines among older adult women reported high compliance with baseline investigations, including glucose, HbA1c, lipid profile, electrolytes, renal and liver function tests, thyroid function tests, prolactin levels, and ECG monitoring [9]. The contrast with our findings suggests that healthcare financing may influence the implementation of guideline-recommended monitoring practices.

Within this study setting, risperidone, chlorpromazine, and haloperidol were among the most frequently prescribed antipsychotics, largely due to their availability and affordability [20]. These medications are commonly associated with hyperprolactinemia secondary to dopamine D2 receptor blockade [21]. In line with the audit standard, 95 patients were expected to undergo baseline prolactin testing; however, no documented baseline prolactin measurements were identified during the two-year study period. Although the high cost of prolactin assays and the absence of local monitoring protocols within the study setting may partly explain this finding, a review of the EMRs also showed that prolactin testing had been requested for some non-first-contact patients who presented with galactorrhea and other suspected prolactin-related adverse effects [22]. This suggests that testing was performed reactively in response to symptoms rather than routinely at treatment initiation. In keeping with our findings, Ghahramani and Bellon revealed that routine prolactin monitoring has not been widely adopted as standard practice and that prolactin is often measured only when patients become symptomatic, but they suggested that a baseline prolactin level should often be collected prior to the commencement of antipsychotics, and a follow-up level after titration is completed. Although this report acknowledged the additional cost of running baseline prolactin, it emphasized the greater benefits to patients over the financial burden [23].

Higher compliance was reported among patients receiving antipsychotic monotherapy, compared with those receiving benzodiazepine or antidepressant monotherapy. This difference was statistically significant and may reflect differences in perceived clinical risk. Clinicians may be more familiar with the adverse effect profile of antipsychotics and, consequently, place greater emphasis on baseline monitoring in this group [22]. Similarly, baseline investigations may have been prioritized for inpatients over outpatients. The significantly higher compliance observed among inpatients may be attributable to the perception that admitted patients are more clinically unwell or unstable, necessitating closer monitoring [24].

Over the study years, mean compliance between 2024 and 2025 only showed minimal improvement (37.41%-41.72%). Although this improvement was not demonstrated to be statistically significant, it may likely suggest early effects of departmental changes following the addition of consultant-level staff and the introduction of academic and clinical meetings. Notwithstanding, the persistence of low overall compliance indicates that barriers, such as the cost of investigations and the lack of fully embedded standard operating procedures, remain significant obstacles to achieving the clinical audit benchmark in this study.

Implications and future research

The persistently low uptake of laboratory and ECG monitoring points to ongoing practical and system-level barriers in routine psychiatric care. Future research should take a closer look at why this gap exists in this setting, particularly the role of cost and the differences between inpatient and outpatient care, and also test practical, locally feasible strategies to improve baseline monitoring in low-resource environments.

Strengths

A major strength of this study is the inclusion of all eligible first-contact patients managed over a two-year period, reducing selection bias and providing a comprehensive assessment of routine psychiatric practice. In addition, the audit employed standardized criteria derived from established NICE and RCPsych recommendations, enhancing the validity of the findings.

Limitations

This study is limited by its retrospective design and reliance on documentation within electronic medical records, which may underestimate true clinical practice. Additionally, the study was conducted in a single tertiary institution, limiting generalizability. Also, this audit did not investigate reasons for noncompliance with recommended investigations, which may arise from a patient or the clinician, limiting the interpretation of the factors underlying poor adherence.

Recommendations

Clinicians should be encouraged to remain up to date with international practice guidelines, such as those issued by NICE, to support the evidence-based monitoring of patients receiving psychotropic medications [7,25-27]. Multidisciplinary collaboration across clinical disciplines within the hospital should also be strengthened to facilitate the development and implementation of local protocols for baseline, routine, and interval monitoring. The routine physical health and laboratory monitoring of patients receiving antipsychotic medications should be prioritized to enable the early detection and management of treatment-related adverse effects. In addition, subsidizing essential baseline investigations, such as prolactin testing and electrocardiography (ECG), may improve patient uptake and adherence to recommended monitoring practices. Finally, a re-audit should be conducted within 6-12 months to evaluate the impact of implemented interventions and monitor progress toward improved compliance with monitoring standards.

## Conclusions

Compliance with recommended baseline physical and laboratory investigations among first-contact patients receiving psychotropic medications was suboptimal, with particularly poor uptake of baseline laboratory and ECG screening. While basic physical assessments were consistently performed, significant gaps remain in guideline-based laboratory and ECG monitoring. Addressing structural barriers, standardizing clinical protocols, and improving the affordability of investigations are essential to improving patient safety and aligning practice with international standards.

## Appendices

Table 8 shows the list of baseline investigations developed from NICE and RCPsych guidelines.

**Table 8:** List of Baseline Investigations Developed From NICE and RCPsych Guidelines Developed based on clinical practice recommendations issued by the National Institute for Health and Care Excellence (NICE) and the Royal College of Psychiatrists (RCPsych) Prescribing Observatory for Mental Health (POMH) guidelines for the baseline monitoring of psychotropic medications [7,8,25-27] BMI, body mass index; HbA1c, glycated hemoglobin; FBC, full blood count; LFTs, liver function tests; E/U/Cr, electrolytes/urea/creatinine; QTc, corrected QT; ECG, electrocardiogram; EEG, electroencephalogram

Section	Category	Investigations/Recommendations
General principles	Timing (inpatients and outpatients), clinical principle, and special considerations	1) Routine baseline investigations within 24-72 hours of admission. 2) For outpatient monitoring, baseline investigations should be done at initial assessment and completed prior to or shortly after the initiation of treatment. 3) Investigations should be individually tailored based on patient factors and clinical judgment. 4) Additional investigations may be required in clozapine/lithium use, comorbidities, organic pathology, and special populations (children and elderly). 5) Include consideration for cardiovascular disease, substance use disorders, traumatic brain injury (TBI), delirium, and neurocognitive disorders
Psychotropic medication-based investigations	Antipsychotics (general)	Weight/BMI, waist circumference, pulse, blood pressure, fasting glucose or HbA1c, lipid profile, prolactin, movement disorder assessment, nutritional and lifestyle assessment, FBC, LFTs, E/U/Cr, and ECG (QTc)
Psychotropic medication-based investigations	Clozapine (additional monitoring)	FBC with differential, ECG, troponin, and C-reactive protein (CRP)
Mood stabilizers	Lithium	Serum lithium levels (12 hours post-dose; weekly until stable), E/U/Cr, thyroid function tests, calcium, weight/BMI, ECG (if >40 years or cardiac risk), and pregnancy test where applicable
Mood stabilizers	Valproate	LFTs, FBC, weight/BMI, and pregnancy test (where applicable)
Mood stabilizers	Carbamazepine	FBC, LFTs, E/U/Cr, and ECG (especially in older adults)
Antidepressants	Standard baseline	Weight/BMI, blood pressure, electrolytes (especially sodium in older adults), and ECG if cardiac risk/QT-prolonging agents or older age
Sedatives and benzodiazepines	Baseline assessment	No routine laboratory tests required; assess respiratory risk, substance use, and comorbid conditions
Stimulants	Baseline assessment	Blood pressure, pulse, weight/BMI, cardiac history, and ECG if cardiac risk factors
Comorbidity-based investigations	Substance use disorders	Urine drug testing and blood alcohol concentration
Comorbidity	Cardiovascular conditions	Electrocardiogram (ECG)
Comorbidity	Organic/neurological conditions	Neuroimaging for traumatic brain injury (TBI), stroke, and organic brain disorders; EEG for seizure disorders or complex partial seizures

Table 9 shows the audit scoring system derived from baseline investigation guidelines for psychotropic medication monitoring.

**Table 9:** Audit Scoring System Derived From Baseline Investigation Guidelines for Psychotropic Medication Monitoring Audit scoring system was adapted from NICE and RCPsych recommendations. Some criteria were modified by consensus of the study team to improve applicability to local psychiatric practice, where guideline recommendations were conditional, medication-specific, or not explicitly stated [7,8,25-27] BP, blood pressure; HbA1c, glycated hemoglobin; E/U/Cr, electrolytes/urea/creatinine; NICE, National Institute for Health and Care Excellence; RCPsych, Royal College of Psychiatrists

Section	Component	Description/Recommendations
Scoring methodology	Binary scoring system	Each completed investigation is assigned a score of 1 and 0 if not completed and no justification for omission is documented
Scoring methodology	Compliance formula	Compliance percentage = (total score obtained ÷ maximum obtainable score) × 100
Scoring methodology	Compliance categories	High compliance (≥75%), moderate compliance (50%-74%), and low compliance (<50%)
Special considerations (add where applicable)	Substance use disorders	Urine drug testing (UDT)
Special considerations (add where applicable)	Alcohol-related disorders	Liver function tests (LFTs)
Special considerations (add where applicable)	Cardiovascular conditions	Electrocardiogram (ECG)
Special considerations (add where applicable)	Neurological conditions	CT/MRI of the brain for traumatic brain injury, stroke, and organic pathology
Special considerations (add where applicable)	Seizure disorders	Electroencephalogram (EEG)
Mood stabilizers	Core investigations	Full blood count (FBC), E/U/Cr, liver function tests (LFTs), ECG, weight, and pregnancy test (mandatory for sodium valproate where applicable)
Sedatives (benzodiazepines and related drugs)	Core investigations	FBC, E/U/Cr, LFTs, and fasting blood sugar (FBS)
Antipsychotics	Core investigations	FBC, E/U/Cr, LFTs, ECG, fasting blood sugar/HbA1c, lipid profile, weight, vital signs (BP and pulse), prolactin level, and assessment of movement disorders
Antidepressants	Core investigations	FBC, E/U/Cr, LFTs, ECG, weight, and vital signs (BP and pulse)
